# GLUT5-overexpression-related tumorigenic implications

**DOI:** 10.1186/s10020-024-00879-8

**Published:** 2024-08-06

**Authors:** Nikola Hadzi-Petrushev, Radoslav Stojchevski, Anastasija Jakimovska, Mimoza Stamenkovska, Slavica Josifovska, Aleksandar Stamatoski, Iliyana Sazdova, Ramadan Sopi, Andre Kamkin, Hristo Gagov, Mitko Mladenov, Dimiter Avtanski

**Affiliations:** 1 Institute of Biology, Faculty of Natural Sciences and Mathematics, Ss. Cyril and Methodius University, Skopje, 1000 North Macedonia; 2 Friedman Diabetes Institute, Lenox Hill Hospital, Northwell Health, 110 E 59th Street, New York, NY 10022 USA; 3 Feinstein Institutes for Medical Research, Manhasset, NY 11030 USA; 4 Donald and Barbara Zucker School of Medicine at Hofstra/Northwell, Hempstead, NY 11549 USA; 5 Faculty of Dental Medicine, University Clinic for Maxillofacial Surgery in Skopje, Ss. Cyril and Methodius University, Skopje, 1000 North Macedonia; 6 Department of Animal and Human Physiology, Faculty of Biology, Sofia University ‘St. Kliment Ohridski’, Sofia, 1504 Bulgaria; 7 Faculty of Medicine, University of Prishtina, Prishtina, 10 000 Kosovo; 8 Institute of Physiology of the Federal State Autonomous Educational Institution of Higher Education “N.I. Pirogov Russian National Research Medical University” Ministry of Health, Moscow, Russian Federation

**Keywords:** GLUT5, Fructose, Tumorigenesis, Pharmacokinetics, Nano-formulation

## Abstract

Glucose transporter 5 (GLUT5) overexpression has gained increasing attention due to its profound implications for tumorigenesis. This manuscript provides a comprehensive overview of the key findings and implications associated with GLUT5 overexpression in cancer. GLUT5 has been found to be upregulated in various cancer types, leading to alterations in fructose metabolism and enhanced glycolysis, even in the presence of oxygen, a hallmark of cancer cells. This metabolic shift provides cancer cells with an alternative energy source and contributes to their uncontrolled growth and survival. Beyond its metabolic roles, recent research has unveiled additional aspects of GLUT5 in cancer biology. GLUT5 overexpression appears to play a critical role in immune evasion mechanisms, which further worsens tumor progression and complicates therapeutic interventions. This dual role of GLUT5 in both metabolic reprogramming and immune modulation highlights its significance as a potential diagnostic marker and therapeutic target. Understanding the molecular mechanisms driving GLUT5 overexpression is crucial for developing targeted therapeutic strategies that can disrupt the unique vulnerabilities of GLUT5-overexpressing cancer cells. This review emphasizes the complexities surrounding GLUT5’s involvement in cancer and underscores the pressing need for continued research to unlock its potential as a diagnostic biomarker and therapeutic target, ultimately improving cancer management and patient outcomes.

## Introduction

### Structure, function, and regulation of GLUT5

The GLUT transporter family, which consists of 14 members, is essential for the efficient diffusion of glucose and other hexoses across cell membranes. These transporters are classified into three types based on their sequence similarity and substrate selectivity (Manolescu et al. 2007). Class I includes GLUT1-4 and GLUT14, which are principally important for glucose transport. Class II, which includes GLUT5, GLUT7, GLUT9, and GLUT11, is known for its fructose transport capabilities (Manolescu et al. 2007; Barron et al. 2016). GLUT5 is an isoform of the glucose transporter family, exclusively found in mammalian cells. This membrane-bound protein is predominantly located in the small intestine, facilitating the selective transport of fructose (Mora and Pessin 2013). Featuring 12 transmembrane domains with intracellular N- and C-termini, GLUT5 diverges from other GLUT isoforms through its specialized role in fructose translocation across the enterocytes’ apical membrane without effectively transporting glucose, thereby exhibiting a unique specificity for fructose (Mora and Pessin 2013; Douard and Ferraris 2008). Despite the comprehensive understanding of other GLUT isoforms, such as GLUT1 and GLUT4, the regulation of GLUT5 expression and its activity remains underexplored (Douard and Ferraris 2008). Dietary fructose has been identified as a modulator of GLUT5 expression in the small intestine, highlighting a regulatory mechanism distinct from the insulin-dependent modulation observed in GLUT4 (Douard and Ferraris 2008). GLUT5’s function in dietary fructose absorption and the distinct metabolic pathways of fructose, primarily within the liver, link excessive fructose consumption to a spectrum of metabolic complications, including insulin resistance, fatty liver disease, and obesity (Barone et al. 2009). The pharmacological exploration of GLUT5 regulation and fructose metabolism is crucial for identifying dietary interventions and therapeutic strategies to mitigate metabolic diseases associated with fructose intake (Shi et al. 2021). Moreover, the potential of GLUT5 inhibitors as therapeutic agents for conditions such as colorectal cancer (CRC) is under investigation, emphasizing the necessity for further research to clarify their efficacy and mechanism of action (Włodarczyk et al. 2021). Ongoing studies in the regulation and pharmacological targeting of GLUT5 are expected to reveal a new understanding of its metabolic functions and therapeutic potential.

### Role of enzyme phosphorylation of fructose and its metabolites

Fructose metabolism within cells is a critical process influenced by various enzymes, primarily through phosphorylation reactions. This metabolic pathway contributes to energy production and plays a significant role in cancer development. Two key enzymes involved in the phosphorylation of fructose and its derivatives are ketohexokinase (KHK) and hexokinase 2 (HK2).

#### Ketohexokinase (KHK)

**Fig. 1:**
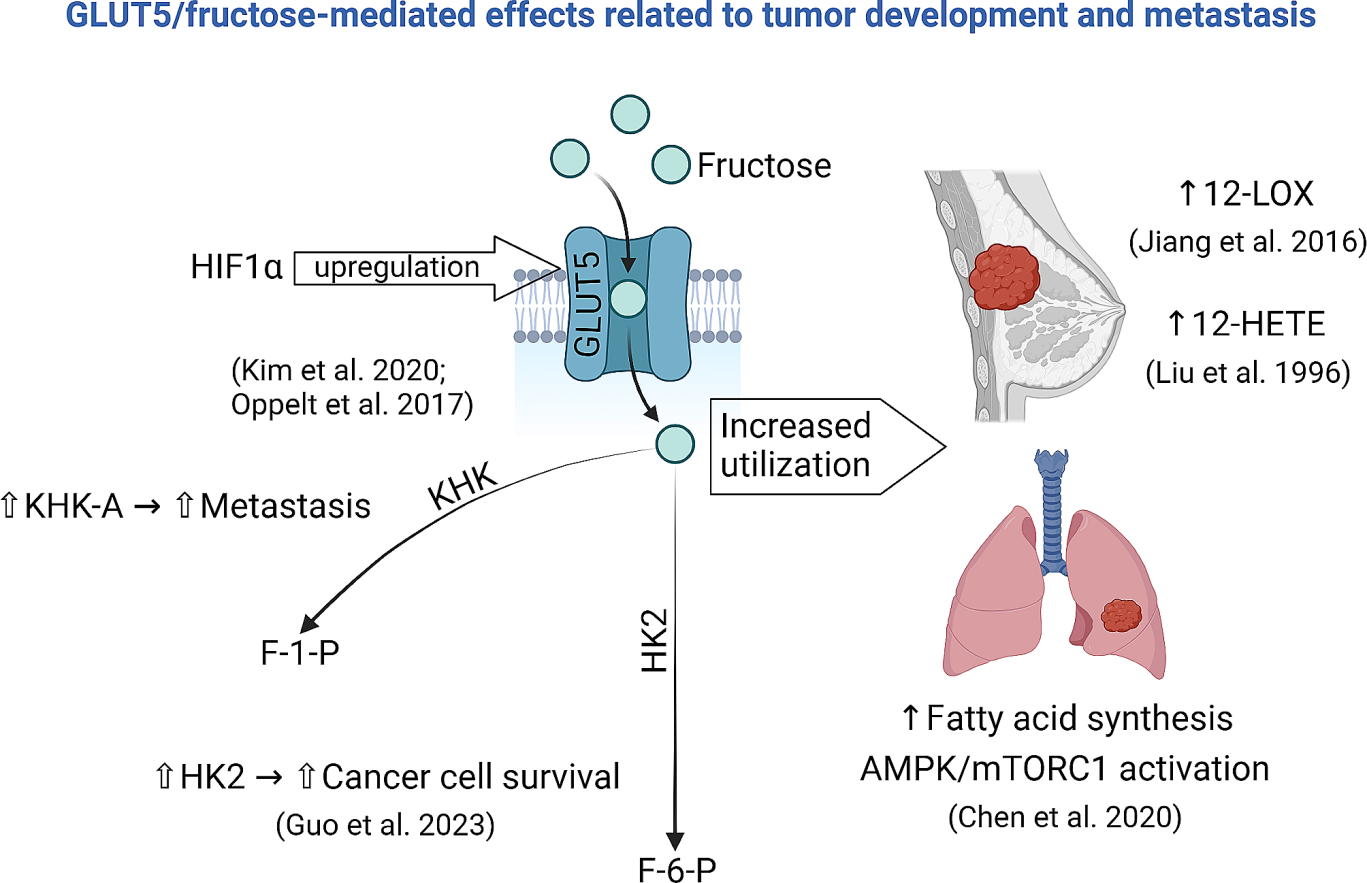
Experimental evidence supports specific GLUT5-mediated effects related to tumor development and metastasis. GLUT5, glucose transporter 5; HIF1α, hypoxia-inducible factor 1-alpha; KHK, ketohexokinase; F-1-P, fructose-1-phosphate; HK2, hexokinase 2; F-6-P, fructose-6-phosphate; 12-LOX, 12-lipoxygenase; 12-HETE, 12-Hydroxyeicosatetraenoic acid; AMPK/mTORC1, AMP-activated protein kinase/mechanistic target of rapamycin complex 1. Created with BioRender.com

KHK is responsible for the phosphorylation of fructose to fructose-1-phosphate (Fig. 1). This enzyme exists in two isoforms, KHK-A and KHK-C. KHK-A, besides its lower affinity for fructose, has been involved in cancer progression through mechanisms beyond its catalytic activity. KHK-A acts as a protein kinase, mediating the phosphorylation of target proteins crucial for cancer cell invasion and metastasis (Kim et al. 2020). The same group demonstrated that KHK-A is predominantly expressed in various cancers, including breast cancer, and is associated with enhanced metastasis. Specifically, KHK-A promotes breast cancer metastasis by facilitating the nuclear translocation and subsequent phosphorylation of tyrosine 3-monooxygenase/tryptophan 5-monooxygenase activation protein eta (YWHAH) at Ser25, which recruits snail family transcriptional repressor 2 (SNAI2) to repress cadherin 1 (CDH1) expression, a key step in epithelial-mesenchymal transition (EMT) (Kim et al. 2020).

In breast cancer, KHK-A levels are significantly higher in metastatic tissues compared to non-metastatic ones, indicating its role in cancer progression (Oppelt et al. 2017). Similarly, KHK expression has been observed in brain regions, suggesting its potential involvement in brain cancers (Oppelt et al. 2017). High dietary fructose intake upregulates KHK activity in the brain, further supporting the enzyme’s role in fructose metabolism and possibly cancer.

#### Hexokinase 2 (HK2)

HK2 catalyzes the phosphorylation of glucose and, to a lesser extent, fructose, forming fructose-6-phosphate. HK2’s role in cancer is well-documented, with high expression levels correlating with poor prognosis in various cancers (Guo et al. 2023). HK2 contributes to the glycolytic pathway and interacts with mitochondrial proteins to prevent apoptosis, promoting cancer cell survival. Although less directly involved in fructose metabolism than KHK, HK2’s broad role in phosphorylating hexose sugars underscores its significance in cancer cell metabolism and survival (Guo et al. 2023).

Based on the presented relations between GLUT5, fructose metabolism, and cancer, two potential therapeutic strategies arise:

(i) *Targeting GLUT5 overexpression alone*: Small molecules developed to bind to GLUT5 can either be transported through it or block its function. These molecules do not directly interact with intracellular enzymes but can inhibit fructose uptake, thereby starving cancer cells that rely on fructose for energy and growth. This approach is particularly relevant in cancers with high GLUT5 expression, where blocking fructose uptake can disrupt cancer cell metabolism and inhibit tumor growth.
(ii) *Utilizing fructose uptake and metabolism in cancer cells*: Beyond targeting GLUT5, understanding the intracellular metabolism of fructose is crucial. The phosphorylation of fructose by KHK and HK2 represents a significant step in fructose metabolism. Targeting these enzymes can disrupt the metabolic pathways that cancer cells exploit for survival and proliferation. For example, inhibiting KHK can prevent the formation of fructose-1-phosphate, thereby blocking a key metabolic route. Similarly, targeting HK2 can affect glucose and fructose metabolism, making it a broad-spectrum strategy against cancer cells.

By addressing both the transport and metabolic aspects of fructose utilization in cancer cells, we can develop comprehensive therapeutic strategies that target multiple points in the fructose metabolism pathway. This dual approach has the potential to enhance the efficacy of cancer treatments by simultaneously inhibiting nutrient uptake and disrupting metabolic processes essential for cancer cell survival.

### Role of HIF1α as a master regulator for GLUT5 expression

Hypoxia-inducible factor 1-alpha (HIF1α) is a transcription factor that plays a crucial role in the cellular response to hypoxia. It is known to regulate the expression of various genes involved in metabolic adaptation, including those encoding glucose and fructose transporters. Recent studies have highlighted HIF1α as a key regulator of GLUT5 expression, linking it to the metabolic reprogramming observed in cancer cells.

#### HIF1α and GLUT5 expression

Under hypoxic conditions, HIF1α is stabilized and translocates to the nucleus, where it binds to hypoxia-responsive elements (HREs) in the promoter regions of target genes. This binding leads to the transcriptional activation of genes involved in glucose and fructose metabolism, including GLUT5. The regulation of GLUT5 by HIF1α is not limited to specific cancer types but represents a more general phenomenon of metabolic adaptation to hypoxia. This regulatory mechanism allows cancer cells to increase fructose uptake, supporting their metabolic needs and promoting survival under low oxygen conditions (Kim et al. 2020; Oppelt et al. 2017).

#### HIF1α in cancer metabolism

The role of HIF1α extends beyond the regulation of GLUT5. It orchestrates a wide array of metabolic processes that enable cancer cells to thrive in hypoxic environments. By upregulating enzymes and transporters involved in glycolysis, lactate production, and fructose metabolism, HIF1α enhances the metabolic flexibility of cancer cells (Fig. 1). This metabolic reprogramming is crucial for the rapid proliferation and survival of cancer cells, making HIF1α a central player in cancer metabolism (Kim et al. 2020; Oppelt et al. 2017).

#### Implications for cancer therapy

Targeting HIF1α and its downstream pathways presents a promising therapeutic strategy. Inhibiting HIF1α activity can disrupt the expression of GLUT5 and other key metabolic regulators, impairing the metabolic adaptability of cancer cells. This approach can sensitize cancer cells to hypoxic stress and reduce their growth and metastatic potential (Kim et al. 2020; Oppelt et al. 2017).

## Tumorigenic implications

### Breast cancer

Breast cancer (BC), a major health concern worldwide, disproportionately affects women but is also seen in men. Jiang et al. (2016b), have exposed a significant association between the overconsumption of fructose/sucrose and an increased risk of BC initiation and progression. BC cell lines are distinguished by their higher fructose consumption compared to normal cells when glucose is absent, indicating fructose’s essential role in cancer cell growth (Monzavi-Karbassi 2010; Gowrishankar et al. 2011). Moreover, fructose is implicated in promoting cancer cell invasion and migration through the upregulation of lipoxygenase-12 (12-LOX) and the production of 12-hydroxy-5Z,8Z,10E,14Z-eicosatetraenoic acid (12-HETE), a key fatty acid in cell membrane function and signaling (Jiang et al. 2016b). The exact mechanism through which fructose activates 12-LOX and its consequent impact on BC remains a topic for further research, with findings suggesting that an increase in 12-HETE levels correlates with heightened tumor cell invasiveness (Liu et al. 1996) (Fig. 1).

Research has underscored GLUT5’s crucial involvement in BC’s fructose-mediated development and metastasis, documenting its high expression levels in BC cell lines and tissues, in contrast to its low presence in the normal mammary epithelium (Zamora-León et al. 1996; Fan et al. 2017; Godoy et al. 2006; Hamann et al. 2018; Wuest et al. 2018) (Table 1). Suppressing GLUT5 using antisense oligonucleotides has proven effective in inhibiting BC cell proliferation (Chan et al. 2004) (Table 1; Fig. 3). Furthermore, it has been shown that hypoxia-inducible factor 1 alpha (HIF1α) plays a role in elevating GLUT5 expression under the hypoxic conditions prevalent within tumor microenvironments (Hamann et al. 2018). Yet, the study by Gowrishankar et al. (2011), challenges the critical nature of GLUT5 in fructose uptake for BC, suggesting the involvement of other transporters and positioning fructose as a marker for detecting cancerous cells.

**Table 1:** Principal roles of GLUT5 in various cancers

Cancer type	Principal role of GLUT5	References
Breast Cancer (BC)	High expression in BC cells and tissues, promoting fructose uptake and enhancing cell proliferation and metastasis. Targeting GLUT5 can inhibit BC cell proliferation.	Zamora-León et al. 1996; Fan et al. 2017; Godoy et al. 2006; Hamann et al. 2018; Wuest et al. 2018; Chan et al. 2004
Colorectal Cancer (CRC)	Upregulated in CRC cells, facilitating fructose-driven glycolysis and tricarboxylic acid cycle. GLUT5-KHK axis plays a role in malignant metabolism and metastasis. Targeting GLUT5 can impede CRC progression.	Mahraoui et al. 1992; Mesonero et al. 1995; Shen et al. 2022; Lin et al. 2021
Prostate Cancer (CaP)	Significant GLUT5 expression in CaP cells, enhancing proliferative and invasive capabilities. GLUT5-mediated fructose uptake contributes to CaP’s aggressive nature.	Reinicke et al. 2012; Carreño et al. 2021; Echeverría et al. 2024
Glioma	Elevated GLUT5 expression in glioma cells and tissues, promoting fructose utilization, cell growth, and tumor progression. GLUT5 is a prognostic marker for gliomas.	Su Li and Gao 2018; Sasaki et al. 2004
Cholangiocarcinoma (CCA)	Overexpressed in CCA cells and tissues, enhancing fructose uptake and glycolysis. GLUT5 silencing reduces cell proliferation and invasion.	Suwannakul et al. 2022
Leukemia	Upregulated in acute myeloid leukemia (AML) cells, facilitating fructose uptake and glycolytic metabolism. GLUT5 inhibition suppresses AML cell proliferation.	Chen et al. 2016
Ovarian Carcinoma (OC)	Higher GLUT5 expression in OC cells and tissues, supporting fructose metabolism and tumor growth. Targeting GLUT5 reduces OC cell proliferation.	Jin et al. 2019
Lung Carcinoma (LC)	Upregulated in lung adenocarcinoma (LUAD) cells, promoting fructose utilization and tumor growth. GLUT5-mediated metabolism supports LC progression.	Weng et al. 2018a, b; Chen et al. 2020
Renal Carcinoma	Elevated GLUT5 expression in clear cell renal cell carcinoma (ccRCC), promoting fructose uptake and cell proliferation. GLUT5 inhibition induces apoptosis in ccRCC cells.	Jin et al. 2019
Intestinal Cancers	Higher GLUT5 levels in intestinal tumors, facilitating fructose-driven tumor growth and metabolic adaptations. Targeting GLUT5 can inhibit tumor growth.	Goncalves et al. 2019

**Fig. 2:**
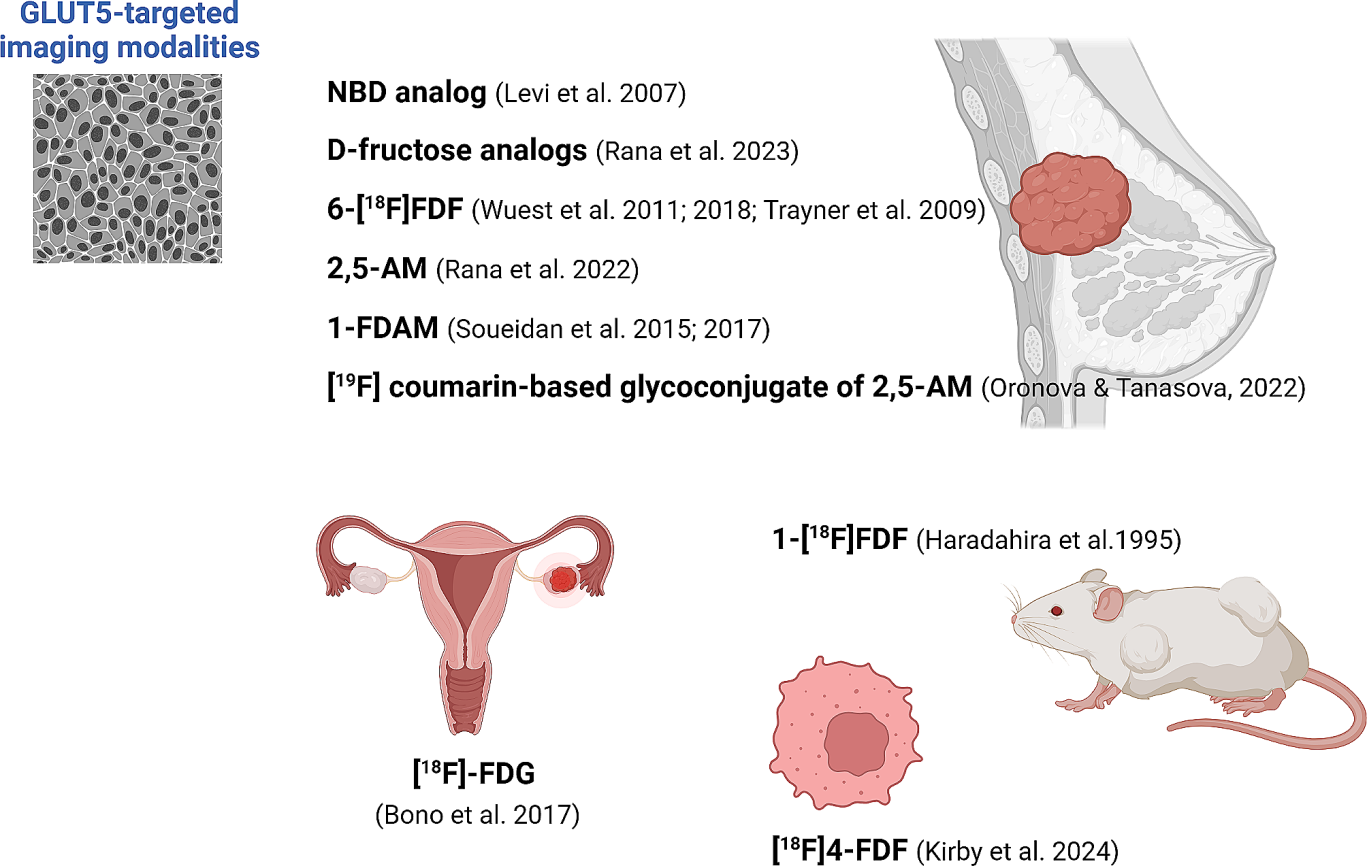
GLUT5-targeted imaging modalities. Various radiotracers and fluorescently labeled probes have been experimentally explored for targeting GLUT-5 in cancer imaging. NBD, 7-nitro-1,2,3-benzoxadiazole; 6-[^18^F]FDF, [^18^F]-labeled 6-deoxy-6-fluoro-D-fructose. 2,5-AM, C-3-modified 2,5-anhydromannitol; 1-FDAM, 1-fluoro-2,5-anhydro-D-mannitol; [^18^F]-FDG, [¹⁸F]Fluorodeoxyglucose; 1-[^18^F]FDF, 1-Deoxy-1-[^18^F]Fluoro-D-Fructose; [^18^F]4-FDF, [^18^F]4-fluoro-4-deoxyfructose. Created with BioRender.com

This new understanding has led to increased interest in targeting GLUT5 for BC imaging and therapy, offering promising pathways for clinical advancements. Adopting fluorescently labeled molecules as an alternative to radiotracers provides a less expensive and safer option for imaging, particularly suited to laboratory studies (Choy et al. 2003). For instance, the creation of a 7-nitro-1,2,3-benzoxadiazole (NBD)-labeled fructose analog by Levi et al. (2007), has enabled the tracking of fructose uptake in BC cell lines through fluorescence microscopy (Fig. 2). Additional research has shown the effective transportation of various fluorescent fructose analogs by GLUT5, shedding light on the metabolic behavior of BC cells (Kannan et al. 2018; Tanasova et al. 2013). Rana et al. (2023) utilized a fluorescent assay to assess the inhibition of GLUT5 uptake by D-fructose analogs, establishing a basis for the design of selective GLUT5 probes (Fig. 2).

The utilization of GLUT5-specific radiotracers within positron emission tomography (PET) and single-photon emission computed tomography (SPECT) enhances molecular imaging of BC cells with improved deep-tissue penetrability and potential for clinical use. Notably, [^18^F]-labeled 6-deoxy-6-fluoro-D-fructose (6-[^18^F]FDF) has been employed in PET imaging to visualize BC, leveraging GLUT5’s role in mediating the transport of 6-[^18^F]FDF and its consequent accumulation in BC cell lines (Wuest et al. 2011, 2018; Trayner et al. 2009) (Fig. 2). The feasibility of employing 6-[^18^F]FDF as a PET radiotracer in clinical settings is further supported by developments in automated synthesis processes and dosimetry calculations (Bouvet et al. 2014). Haradahira et al. (1995) reported that 1-Deoxy-1-[^18^F]Fluoro-D-Fructose (1-[^18^F]FDF) was synthesized by nucleophilic substitution of [^18^F]fluoride ion (Fig. 2). The tissue distributions in rats and tumor-bearing mice showed initial high uptake and subsequent rapid washout of the radioactivity in the principal sites of D-fructose metabolism (kidneys, liver, and small intestine). The uptakes in the brain and tumor (fibrosarcoma) were the lowest and moderate, respectively, but tended to increase with time. Metabolic studies indicated that the fluorinated analog remained unmetabolized in the brain and tumor tissues, suggesting it does not undergo metabolic trapping without appreciable organ or tumor-specific localization.

In recent developments, Kirby et al. (2024) explored the potential of [^18^F]4-fluoro-4-deoxyfructose ([^18^F]4-FDF) as a novel radiotracer for PET imaging. Their study demonstrated that [^18^F]4-FDF effectively accumulates in tumors with minimal bone uptake, contrasting with [^18^F]6-FDF, which shows significant bone uptake due to metabolic processing. [^18^F]4-FDF exhibited low uptake in healthy brain and heart tissues, typically high in glycolytic activity, making it a promising tool for mapping neuro- and cardio-inflammatory responses. The metabolic tracing indicated that [^18^F]4-FDF gets trapped as fluorodeoxyfructose-1-phosphate within the cell, unlike the C1 and C6 radio analogs, thus allowing for effective imaging of inflammation and potentially cancer (Fig. 2).

**Fig. 3:**
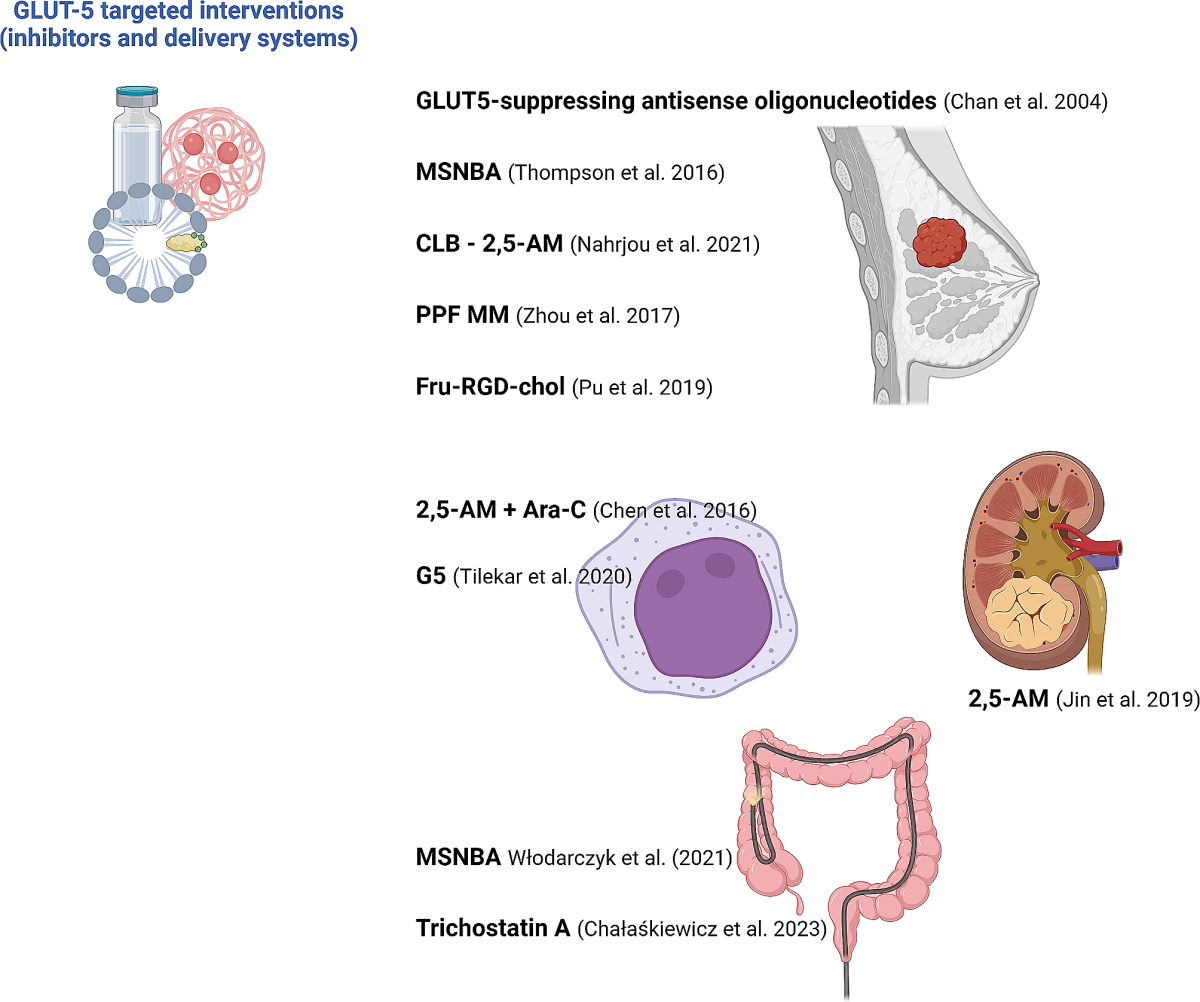
GLUT5-targeted interventions. Promising experimental data reveal the potential application of various drug constructs targeting the GLUT5 transporter in different types of cancer. MSNBA, N-[4-(methylsulfonyl)-2-nitrophenyl]-1,3-benzodioxol-5-amine; CLB − 2,5-AM, chlorambucil (CLB) 2,5-anhydro-D-mannitol conjugate; PPF MM, poly(ε-caprolactone)-polyethylene glycol (PCL-PEG-Fru)/D-α-tocopheryl polyethylene glycol 1000 succinate (TPGS) mixed micelles; Fru-RGD-chol, fructose-arginylglycylaspartic acid (RGD)-cholesterol liposome; 2,5-AM + Ara-C, 2,5-anhydro-D-mannitol + cytosine arabinoside; G5, 2-[5-(4-Chlorobenzylidene)-2,4-dioxothiazolidin-3-yl]-N-(4-chloro-2-trifluromethylphenyl) acetamide. Created with BioRender.com

The proposal to use radiolabeled C-3-modified 2,5-anhydro-D-mannitol (2,5-AM) compounds for BC molecular imaging via PET presents another promising method (Rana et al. 2022). Earlier work by Soueidan et al. has demonstrated the synthesis and evaluation of 1-deoxy-1-fluoro-2,5-anhydro-D-mannitol (1-FDAM) as a potential imaging agent, highlighting its transport into BC cell lines via GLUT5 and suggesting routes for developing new molecular imaging probes (Soueidan et al. 2015; 2017) (Fig. 2). In a particular study, Nahrjou et al. (2021), explored the feasibility of delivering the bioactive anticancer agent chlorambucil (CLB) via the GLUT5 transporter using a 2,5-anhydro-D-mannitol conjugate, aiming to achieve cancer-specific cytotoxicity (Fig. 3). These conjugates exhibit enhanced selective cytotoxicity towards BC cells (MCF-7 and MDA-MB-231), compared to non-cancerous breast cells (184B5) due to higher GLUT5-mediated uptake in cancer cells (Nahrjou et al. 2021). Additionally, a significant relationship was found between cancer selectivity and the size of the conjugate, with a decrease in GLUT5-mediated uptake correlating with increases in conjugate size and hydrophobicity.

Moreover, the group led by Tanasova has extensively worked on fluorescence-labeled probes for GLUT5, including 2,5-AM and coumarin glycoconjugates. Their studies have shown the feasibility of using these probes for high-throughput cancer identification and the potential development of a novel GLUT5-targeting glycoconjugate as a PET probe (Begoyan et al. 2018; Nahrjou et al. 2021; Oronova & Tanasova 2023) (Fig. 2).

In oncology, targeted therapies have demonstrated superior efficacy and diminished side effects compared to conventional treatment modalities. Preclinical studies focusing on GLUT5-targeted interventions for BC have shown encouraging outcomes. Inhibiting GLUT5 and thus blocking fructose uptake interferes with the metabolic pathways of cancer cells, leading to apoptosis and increased susceptibility to traditional treatments. A significant breakthrough was achieved with the discovery of N-[4-(methylsulfonyl)-2-nitrophenyl]-1,3-benzodioxol-5-amine (MSNBA), a highly potent and specific GLUT5 inhibitor, shown to significantly reduce fructose-driven proliferation in BC cell lines (Thompson et al. 2016) (Fig. 3). The unique selectivity of the GLUT5 transporter has been exploited in developing various delivery systems, such as constructs and micelles imbued with fructose components, designed to specifically deliver chemotherapeutic agents to BC cells (Englert et al. 2017; Zhou et al. 2017; Lu et al. 2017). Zhou et al. (2017) introduced a significant advancement in this area by developing D-fructose-modified poly(ε-caprolactone)-polyethylene glycol (PCL-PEG-Fru) diblock amphiphiles. When combined with D-α-tocopheryl polyethylene glycol 1000 succinate (TPGS) to form PCL-PEG-Fru/TPGS mixed micelles (PPF MM), these nanocarriers demonstrated a promising potential for GLUT5-mediated, cell-specific delivery in cancer therapy (Fig. 3). The construction of PCL-PEG-Fru employs Cu(I)-catalyzed click chemistry, with the resultant PPF MM showcasing notably higher uptake in MCF-7 cells, which overexpress GLUT5, compared to L929 cells, where GLUT5 is not overexpressed. The study further proves that free D-fructose can competitively inhibit the incorporation of PPF MM in MCF-7 cells, underscoring their GLUT5 specificity. In vivo assessments in MCF-7 breast tumor-bearing mice xenografts showed selective tumor accumulation of PPF MM, highlighting their utility as a targeted drug delivery system in cancer therapy.

Targeting efficiency has been improved by integrating additional ligands, including short peptides, biotin, and folic acid, with fructose in complex delivery vehicles like multifunctional liposomes or carbon nanotubes (Pu et al. 2019; Li et al. 2022; Omurtag et al. 2020) (Fig. 3). Pu et al. (2019) pioneered the development of innovative liposomes designed to recognize GLUT5 and integrin *αvβ3*, markers predominantly expressed in triple-negative BC cell lines, including MDA-MB-231 and 4T1. These liposomes were synthesized by covalently bonding fructose and the peptide arginylglycylaspartic acid (RGD) to cholesterol molecules, which were then integrated into the liposome structure, creating Fru-RGD-chol and Fru-RGD-chol variants. Both types of liposomes demonstrated significant cellular uptake in vitro and efficient tumor accumulation in vivo. Notably, the Fru-RGD-chol variant exhibited superior targeting capabilities, making it a promising candidate for targeted drug delivery applications.

Moreover, the implementation of multimodal GLUT5-targeting nanoparticle systems has facilitated a synergistic approach, combining chemotherapy with photothermal and photodynamic therapies for augmented therapeutic impact (Cetin Ersen et al. 2023). Despite these advancements, challenges such as potential off-target effects and unintended consequences on the metabolism of normal tissues necessitate a comprehensive exploration of these strategies prior to clinical application.

The ongoing exploration of these methodologies stresses the dynamic potential of GLUT5-targeted therapy and diagnostics, promising a new horizon in the treatment of GLUT5 overexpression-related diseases.

### Cholangiocarcinoma

**Fig. 4:**
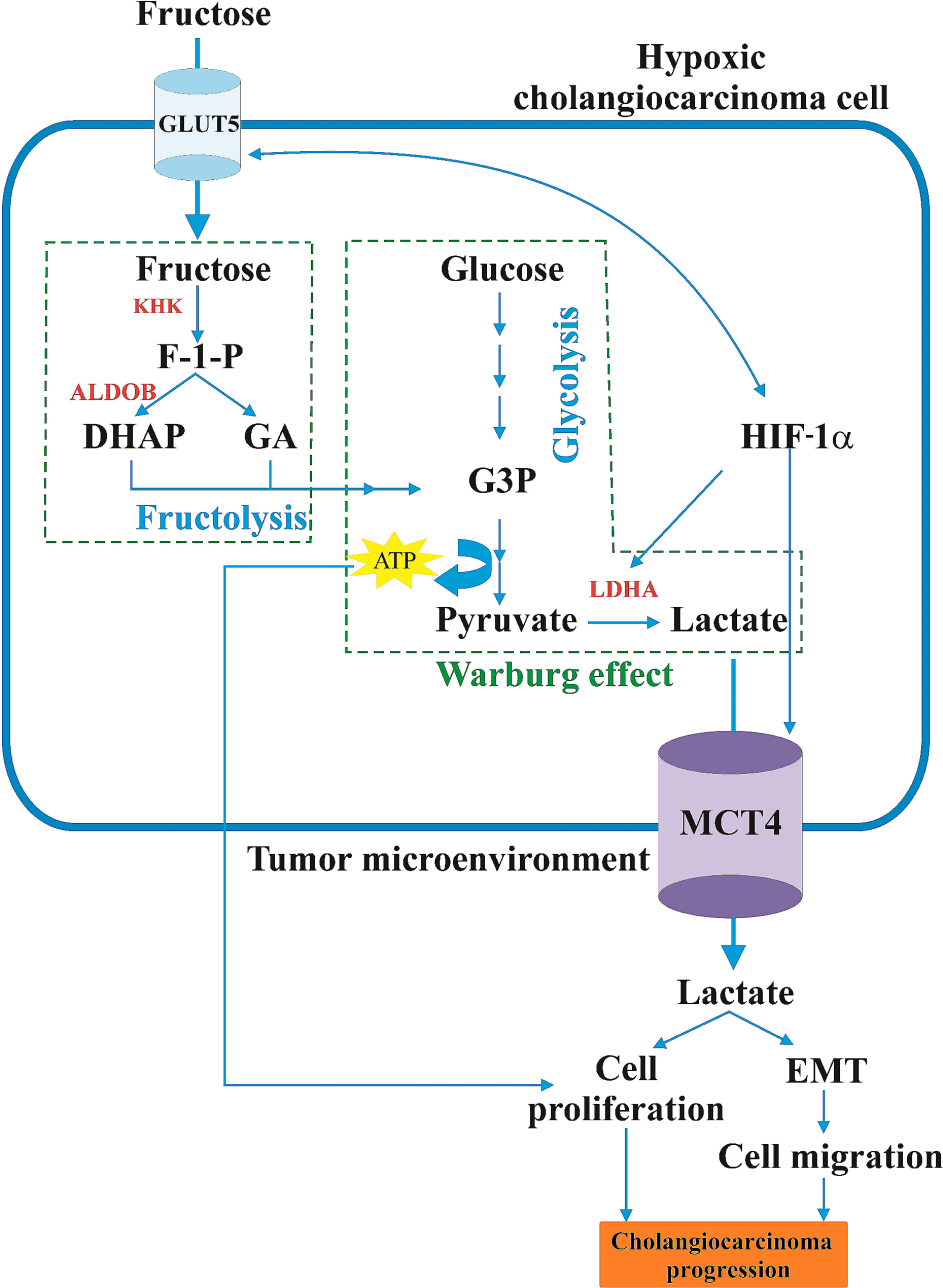
Potential pathway through which GLUT5 contributes to tumorigenesis in CCA cells. KHK, ketohexokinase; ALDOB, aldolase B; LDHA, lactate dehydrogenase; F-1-P, fructose-1-phosphate; DHAP, dihydroxyacetone phosphate; GA, glyceraldehyde; G3P, glyceraldehye-3-phosphate; HIF1α, hypoxia-inducible factor 1 alpha; MCT4, monocarboxylate transporter. Created with BioRender.com

Cholangiocarcinoma (CCA) is a heterogeneous group of malignancies originating from the biliary tract (Banales et al. 2020). It is a silent and aggressive type of cancer with increasing incidence and mortality (Banales et al. 2020; Kirstein and Vogel 2016). Recently, light has been shed on the role of GLUT5 in CCA tumorigenesis, which can potentially lead to an improved diagnosis and treatment. It has been shown that *SLC2A5*, the gene encoding GLUT5, is overexpressed in human CCA cells and tissues compared to normal cholangiocytes and normal liver tissue (Suwannakul et al. 2022) (Table 1). Moreover, in the advanced pathological stages III and IV, the upregulation of GLUT5 is increased compared to the earlier stages I and II (Suwannakul et al. 2022). In the presence of fructose supplementation, CCA cells exhibit higher proliferative rates and adenosine triphosphate (ATP) production than those in glucose-supplemented medium. In addition, it has been shown that fructose-consuming mouse xenografts had increased tumor growth compared to those consuming water (Suwannakul et al. 2022). Taken together, this conveys the idea that GLUT5-mediated fructose uptake may contribute to the progression and growth of CCA. In support of this, it has been shown that GLUT5 silencing attenuates the cell proliferative effect, lowers CCA cell’s fructose uptake, suppresses cell invasion and migration, and reduces tumor growth in mouse xenografts (Suwannakul et al. 2022). GLUT5 silencing has also led to upregulation of the epithelial-like cell marker, E-cadherin, and downregulation of the mesenchymal-like cell marker, N-cadherin, implying that GLUT5 may influence the metastatic potential of CCA cells by regulating epithelial-to-mesenchymal transition (EMT) processes (Suwannakul et al. 2022). The development of CCA involves complex interactions between external signaling molecules in the tumor microenvironment, abnormal activation of cell surface membrane proteins, and deregulations in intracellular signaling pathways (Liu et al. 2023). It has been shown that the upregulation of GLUT5 impacts genes linked to fructose metabolism and the Warburg effect, such as ketohexokinase (*KHK*), aldolase B (*ALDOB*), lactate dehydrogenase A (*LDHA*), and HIF1α (*HIF1α*) (Suwannakul et al. 2022) (Fig. 4). When fructose is taken by the hepatocytes, it is metabolized to fructose-1-phosphate by KHK and then rapidly cleaved to dihydroxyacetone phosphate (DHAP) and glyceraldehyde (GA) by ALDOB (Merino et al. 2019). Both enzymes are overexpressed in CCA due to GLUT5 overexpression (Suwannakul et al. 2022). Cancer cells undergo the Warburg effect to meet the increased demand for ATP, wherein GA and DHAP enter a series of chain reactions leading to pyruvate production (Danhier et al. 2017). In this metabolic process, pyruvate is converted to lactate by LDHA, which is also deregulated in CCA in a GLUT5-expression-dependent manner, indicating that elevated GLUT5 levels result in increased lactate production in cancer cells (Suwannakul et al. 2022; Cai et al. 2013). Additionally, elevated LDHA in bladder cancer is correlated with cancer proliferation and metastasis (Jiang et al. 2016a). Monocarboxylate transporter 4 (MCT4), whose expression is also proportionally influenced by GLUT5 expression in CCA cells, plays a crucial role in transporting the accumulated lactate into the extracellular environment (Suwannakul et al. 2022; de la Cruz-López et al. 2019). This process contributes to extracellular acidification in the tumor microenvironment, thus promoting tumor invasion and metastasis (de la Cruz-López et al. 2019). Accordingly, the inhibition of MCT4 has been shown to reduce tumor proliferation in colorectal cancer (Kim et al. 2018). Lastly, HIF1α plays a key role in cellular adaptation to hypoxia, a common feature of carcinogenesis, and is known to regulate lactate levels by controlling the expression of LDHA and MCT4 (Serganova et al. 2018). The correlation between lactate-MCT/HIF1α and metabolic reprogramming of macrophage polarization in gastric cancer has been observed in interactions between cancer cells and immune cells (Zhang and Li 2020). Additionally, it has been reported that HIF1α regulates GLUT1 and GLUT5 in breast cancer cells and tissues during hypoxia (Hamann et al. 2018).

These findings suggest that GLUT5 upregulation leads to enhanced glycolysis and ATP production through the fructolysis-Warburg pathway. The subsequent increase in lactate production contributes to the acidic tumor microenvironment and thus may promote tumor invasion and metastasis.

### Choriocarcinoma

Choriocarcinoma is an uncommon yet aggressive form of gestational trophoblastic neoplasm that arises from the placenta (Bishop and Edemekong 2023). During pregnancy, focusing on getting enough hexoses among other nutrients, is vital for fetal development and growth. Evidence suggests that high fructose intake during gestation may contribute to adverse effects, including metabolic syndrome in offspring during adulthood (Asghar et al. 2016; Koo et al. 2021). Nonetheless, the association between fructose metabolism and the initiation of choriocarcinoma tumorigenesis remains elusive. Investigations have demonstrated that human choriocarcinoma cells can express the GLUT5 fructose transporter and metabolize fructose, although less efficiently than glucose (Shah et al. 1999). This highlights the need for more extensive research, particularly considering that the expression of GLUT5 in choriocarcinoma cells could signify a significant role for fructose metabolism in the cancer’s development.

### Glioma

Gliomas are the most prevalent primary brain tumors and are characterized by their aggressive metastatic and invasive behavior (Weller et al. 2015; Davis 2018; Qi et al. 2017). Like many cancers, glioma cells undergo metabolic reprogramming, displaying the Warburg effect and a propensity for fructose utilization (Poff et al. 2019; Su et al. 2018) (Table 1). Studies have indicated that gliomas can proliferate and form colonies in fructose-enriched mediums comparably to glucose mediums. However, fructose fails to restore normal proliferation in microglial cells under glucose deprivation. This discrepancy may stem from the differential expression of GLUT5, which is significantly higher in glioma cells and tissues than in normal microglia and surrounding tissues (Su et al. 2018). The suppression of GLUT5 in glioma cells diminishes their proliferation and colony-forming capabilities, underscoring GLUT5’s crucial role in glioma cell growth and tumorigenesis via fructose metabolism (Su et al. 2018). In vivo evidence further supports that GLUT5 silencing substantially impedes tumor progression, highlighting GLUT5’s critical function (Mahraoui et al. 1992). Additionally, research by Su et al. (2018), has linked elevated GLUT5 expression with reduced survival rates, proposing GLUT5 as a prognostic marker for gliomas. Sasaki et al. (2004) complement this by demonstrating that astrocytic gliomas, particularly pilocytic astrocytomas, exhibit a higher density of GLUT5-positive microglia compared to the less malignant oligodendroglial gliomas (Table 1). These insights emphasize the therapeutic and prognostic potential of targeting GLUT5 in glioma treatment strategies.

### Leukemia

Acute leukemias are aggressive hematologic malignancies marked by swift progression and altered glucose metabolism that often display the Warburg effect (Chen et al. 2016; Konończuk et al. 2022; Padda et al. 2021). Acute myeloid leukemia (AML) cells, in particular, exhibit a pronounced glycolytic metabolism. In low-glucose environments, AML cells adapt by increasing fructose intake, facilitated by upregulated GLUT5 expression (Chen et al. 2016; Herst et al. 2010). This adaptation allows AML cells to maintain vigorous proliferation in glucose-deprived conditions (Table 1). Notably, GLUT5 overexpression is prevalent in various AML cell lines and bone marrow blasts of AML models, contrasting with normal monocytes (Chen et al. 2016). Silencing the *SLC2A5* gene in AML cells leads to decreased fructose uptake and inhibited cell proliferation. Conversely, *SLC2A5* overexpression enhances fructose-driven proliferation, colony formation, and cellular mobility. Overexpressed *SLC2A5* in AML also boosts glycolysis byproducts, including pyruvate, lactate, and alanine, highlighting the critical role of fructose in glycolytic flux via GLUT5. Chen et al. (Chen et al. 2016) have shown that high *SLC2A5* expression correlates with poorer outcomes, linking above-median or above-mean levels with reduced survival in AML patients. Research into pharmacological GLUT5 inhibition with 2,5-AM revealed its efficacy in suppressing fructose-driven proliferation and mobility in AML under glucose restriction and its synergistic effects with the chemotherapy drug Ara-C, enhancing treatment potential (Chen et al. 2016) (Fig. 3). Additionally, Tilekar et al. (2020), identified a thiazolidinedione derivative, G5, as a potent GLUT5 inhibitor, exhibiting anti-proliferative actions and promoting cell cycle arrest in leukemia cells, leading to apoptotic and necrotic cell death (Fig. 3). These findings highlight the critical dependency of leukemia cells on fructose metabolism through GLUT5 and position GLUT5 inhibition as a promising approach for leukemia therapy.

### Liver carcinoma

Liver metastases frequently occur in advanced-stage tumors and are associated with significantly worsened prognoses (Bilen et al. 2019). Since the liver is the main site for fructose metabolism, it necessitates a deeper understanding of how fructose transporters, especially GLUT5, are involved in liver carcinogenesis. Excessive fructose intake is linked to numerous health issues related to oxidative stress, inflammation, increased levels of uric acid and triglycerides, hypertension, and insulin resistance, all known risk factors for liver disease development and progression (Muriel et al. 2021). Despite these associations, the expression and role of GLUT5 in liver cancer cells have yet to be fully elucidated. A particular study highlighted that GLUT5 expression is notably higher in metastatic liver tumors compared to normal liver and lung tissues, indicating a significant alteration in metabolic preferences (Kurata et al. 1999). This variation in GLUT5 expression between primary and metastatic liver tumors hints at distinct fructose metabolism pathways, which could play a critical role in the metastatic processes. Intriguingly, evidence from another study indicates that liver metastases from lung cancer specifically show GLUT5 upregulation, suggesting a specialized adaptation that allows these tumors to utilize fructose for energy more effectively in metastatic sites (Kurata et al. 1999).

### Lung carcinoma

Lung carcinoma (LC) ranks as one of the top causes of cancer-related deaths globally due to its aggressive cell proliferation within the lungs (Molina et al. 2008). The development of LC is driven by intricate molecular processes, leading to tumor growth, progression, and metastasis. It is categorized into two main histological subtypes: small-cell lung carcinoma (SCLC) and non-small-cell lung carcinoma (NSCLC), with NSCLC making up about 85% of all LC incidences. This category includes various forms like adenocarcinoma, squamous, and large cell carcinoma. In contrast, SCLC accounts for 15% of lung cancer cases, characterized by rapid growth and significant genetic variability, which complicates therapeutic decision-making (Barta et al. 2019). Despite the introduction of targeted therapies, overcoming immune evasion remains a critical issue, especially in patients without identifiable driver mutations. The five-year survival rate for LC patients is low, highlighting the urgent need for an in-depth understanding of the disease’s fundamental mechanisms (Qin et al. 2016; Hirsch et al. 2017; Chen et al. 2014).

A significant yet underexplored aspect of LC research is the role and overexpression of GLUT5 in LC and its metastases. A study by Kurata et al. (1999) pioneered this investigation, analyzing GLUT family gene expressions, including GLUT5, across primary LC, metastatic liver tumors, and normal lung tissue. Their results demonstrated significantly higher GLUT5 expression in metastatic liver tumors compared to normal tissues, suggesting these tumors’ unique ability to exploit fructose for energy, possibly aiding their aggressive progression (Kurata et al. 1999). Although this study significantly advanced our understanding, it lacked direct comparisons between GLUT5 expression in primary and metastatic lung tumors, leaving a gap in our comprehension of GLUT5’s specific contributions to LC metastasis. Nonetheless, it hints at distinct energy metabolism and pathways in metastatic lung tumors, underscoring the importance of further research into how these processes influence their malignancy and capacity for organ invasion.

Weng et al. (2018a, b) studied the impact of fructose metabolism on lung adenocarcinoma (LUAD) cells, particularly focusing on the mediation by GLUT5 and its role in promoting metabolic activity and tumor growth. Utilizing in vitro analyses with LUAD patient tissue samples, the authors observed a marked upregulation of GLUT5 in NSCLC samples, notably in LUAD, compared to healthy lung tissue (Table 1). GLUT5 upregulation was significantly linked to a poorer prognosis in LUAD patients, suggesting its vital role in the disease’s evolution and aggression (Weng et al. 2018a, b). Despite these findings, the mechanisms governing GLUT5 expression and its precise function within LC contexts remained vague, prompting further investigation into whether LC cells preferentially use fructose via GLUT5 amidst other metabolic substrates in vivo to support their growth and metabolic needs.

Furthermore, Chen et al. (2020) presented results that LC cells preferentially utilize fructose as an alternative to glucose by enhancing GLUT5 expression (Table 1;). Through in-depth in vitro and in vivo studies, they demonstrated that GLUT5 overexpression in LC cells correlates with increased fructose absorption, stimulating fatty acid synthesis and activating the AMPK/mTORC1 signaling pathway (Fig. 1). These metabolic shifts not only facilitate tumor growth but also provide the essential energy and substrates needed for the cancer cells’ rapid proliferation (Chen et al. 2020). The findings emphasize the critical function of fructose metabolism, mediated through GLUT5, in driving LC progression and highlight the potential of targeting GLUT5 as a novel therapeutic approach to combat fructose-dependent cancer cells.

### Oral squamous carcinoma

Oral squamous cell carcinoma (OSCC), ranking as the ninth most prevalent cancer globally, represents a formidable challenge in the field of head and neck oncology (Bray et al. 2018). Treatment modalities for advanced-stage lesions (stages I-III) typically include surgery and radiotherapy, while high-risk patients, identified by extensive lymph node involvement or distant metastases, undergo chemotherapy, as outlined by Reis et al. (2011). In cases of stage IV OSCC, a targeted non-surgical strategy incorporating intensified chemotherapy aims to improve disease outcomes, prolong survival, and enhance life quality for affected individuals. Despite these efforts, for all oral cavity cancers, the 5-year overall relative survival rate hovers around 57%, indicating a plateau in the effectiveness of current treatment approaches over the years (Marsh et al. 2011).

In their in vitro study, Paolini et al. (2022), assessed the expression of GLUT transporters in human OSCC cells and compared these findings with those from normal oral keratinocytes (OKF6), focusing on the effects of GLUT1-specific inhibitors BAY876 and WZB117. This study contributes to our understanding of GLUT transcriptional regulation within OSCC, potentially influencing cancer cell metabolism and highlighting novel therapeutic targets. A key finding was the consistent expression of GLUT5 across all tested cell lines, indicating its role in both normal and malignant oral cell metabolisms. Additionally, the study observed a reduction in GLUT5 mRNA expression in OSCC cells following treatment with BAY876, suggesting a regulatory relationship between GLUT1 and GLUT5 expressions (Paolini et al. 2022). These initial observations provide a valuable foundation for future investigations into the role of GLUT5 in the pathogenesis of OSCC, pointing towards its potential as a therapeutic target.

### Ovarian carcinoma

Ovarian carcinoma (OC) represents a life-threatening form of cancer affecting women, primarily attributed to its pelvic location, asymptomatic early stages, and limited practical diagnostic approaches. Consequently, most OC cases are identified at advanced stages, significantly diminishing survival prospects (Park et al. 2017). Obesity is recognized as a critical risk factor for OC, affecting both its onset and patient outcomes (Tworoger and Huang, 2016). The impact of dietary components on cancer proliferation has become a focal point of research, with high-fructose consumption being investigated for its potential role in cancer initiation (Lyssiotis and Cantley 2013). Despite existing evidence linking fructose intake to tumorigenesis, specific studies on fructose’s relationship with OC and the involvement of GLUT5 remain scarce (Joung et al. 2017).

In their study, Bono et al. (2017), assessed the risk of false-positive results in detecting malignancy via positron emission tomography with ^18^F-fluorodeoxyglucose positron emission tomography-computed tomography (^18^F-FDG PET-CT), particularly concerning benign ovarian tumors, while examining GLUT5 expression’s implication in tumorigenesis (Fig. 2). Investigating solid ovarian tumors flagged as potentially malignant based on magnetic resonance imaging (MRI) and [¹⁸F]Fluorodeoxyglucose [^18^F-FDG] uptake, the research revealed that these tumors exhibited positive ^18^F-FDG signals, leading to a potential misdiagnosis as malignant via PET-CT. However, surgical intervention and subsequent frozen section diagnosis clarified that these instances were benign thecomas. Immunohistochemical analysis further identified immunoreactive GLUT5 expression within these tumor tissues, which indicates its potential involvement in tumor formation and presents it as a candidate for therapeutic targeting (Bono et al. 2017).

Jin et al. (2019) conducted research into fructose metabolism within OC cells, uncovering that these cells sustain growth rates in fructose media lacking glucose, highlighting their efficient fructose utilization compared to non-tumorous ovarian cells’ incapacity for similar growth in fructose environments. Their exploration into GLUT5 expression within OC tissues further aimed to identify correlations with clinicopathological features and patient survival outcomes (Table 1). The study revealed that OC tissues exhibit significantly higher GLUT5 expression than adjacent non-cancerous ovarian tissues, linking elevated GLUT5 levels to advanced tumor stages and poorer prognoses in OC patients (Jin et al. 2019). Further experiments demonstrated that GLUT5 silencing markedly reduces fructose absorption and metabolism, decreasing proliferation, colony formation, and migration in OC cells. This effect was accompanied by the downregulation of critical enzymes in fructose metabolism, such as KHK, ALDOB, and triose kinase (TK). The study also showed that a high-fructose diet accelerates tumor growth in vivo, whereas GLUT5 inhibition reduces tumor expansion in a mouse xenograft model. Consequently, Jin et al. (2019) concluded that GLUT5 is integral to fructose metabolism and the progression of OC, proposing GLUT5 targeting as a viable therapeutic approach.

### Prostate cancer

Prostate cancer (CaP) remains the second most prevalent cause of cancer-related deaths among adult men in the United States, underscoring the importance of identifying and understanding both inherent and modifiable risk factors (Siegel et al. 2019). Recent research has shifted towards modifiable risk factors, such as dietary habits, revealing a direct link between increased consumption of dietary sugar, particularly high fructose corn syrup (HFCS), and an increased risk of developing symptomatic CaP (Makarem et al. 2018). Moreover, fructose’s role in promoting cell proliferation and metastasis in pancreatic cancers has been documented, suggesting its broader implications in cancer pathophysiology (Liu et al. 2010).

A significant investigation into the cellular dynamics of GLUT1 and GLUT5 in both benign and malignant prostate tissues was undertaken by Reinicke et al. (2012). This study aimed to outline the distribution and functional implications of these glucose transporters within the prostate, examining their expression in conditions ranging from high-grade prostatic intraepithelial neoplasia (HGPIN) to clinical cases of CaP (Table 1). Findings indicated a reduced expression of GLUT5, a fructose-specific transporter, in CaP tissues compared to their benign counterparts. This reduction suggests a potential decrease in fructose consumption by CaP cells. However, the presence of GLUT5 in HGPIN lesions implies that fructose metabolism may be crucial for the metabolic needs and survival of precancerous epithelial cells, hinting at a complex relationship between fructose and early-stage prostate carcinogenesis (Reinicke et al. 2012).

Based on this, Carreño et al. (2021), sought to further clarify the role of fructose transporters, particularly GLUT5 and GLUT9, in the context of CaP. Through comprehensive analyses of benign and malignant prostate tissue specimens and CaP cell lines, significant GLUT5 expression was observed in CaP samples, identifying it as a primary mediator of fructose transport in these cells (Table 1). Fructose stimulation was found to enhance the proliferative and invasive capabilities of CaP cells in vitro, with in vivo experiments revealing that dietary fructose significantly fosters tumor growth and cellular proliferation. These findings emphasize the potential of GLUT5-mediated fructose uptake to contribute to the aggressive nature of CaP, presenting a compelling argument for targeting GLUT5 in therapeutic strategies (Carreño et al. 2021).

Echeverría et al. (2024), provided additional insights into the role of GLUT5 in CaP. They also showed that inhibiting GLUT5 reduces tumor growth and metastasis in vivo, supporting the idea that GLUT5 is not only involved in early-stage prostate carcinogenesis but also plays a critical role in the progression and aggressiveness of the disease. These findings further highlight the therapeutic potential of targeting GLUT5 in CaP treatment (Echeverría et al. 2024).

### Renal carcinoma

Renal carcinoma, recognized as a particularly lethal form of cancer, is responsible for a substantial number of cancer-related fatalities globally (Siegel et al. 2017; Barata and Rini, 2017; Garcia and Rini 2007). Despite significant strides in treatment modalities involving targeted therapies and immune checkpoint inhibitors, the prognosis for patients remains unsatisfactory (Curti 2018; Ghatalia et al. 2017; Wong et al. 2017; Quinn and Lara, 2015). A notable barrier to improving patient outcomes is the emergence of chemotherapeutic resistance, highlighting the imperative need for identifying new therapeutic targets (Heinzer et al. 2001).

A study by Jin et al. (2019), explored the implications of GLUT5 in clear cell renal cell carcinoma (ccRCC), exploring its influence on the malignancy’s progression through fructose utilization and its viability as a therapeutic target. This study found a marked elevation in GLUT5 expression within ccRCC tissues and cell lines compared to normal kidney cells, establishing a significant differential in fructose uptake rates. The utilization of fructose by ccRCC cells promoted cell proliferation, colony development, and survival, underscoring the metabolic adaptations fueling the cancer’s growth (Table 1). An increase in GLUT5 expression in ccRCC cells was correlated with an intensified rate of fructose utilization, further propelling cell growth and colony formation. In contrast, *SLC2A5* deletion reduced fructose-driven cell proliferation and increased apoptosis rates among ccRCC cells. Similarly, treatment with the GLUT5 inhibitor, 2,5-AM, suppressed fructose-induced cell growth while stimulating apoptosis in ccRCC cells (Jin et al. 2019) (Fig. 3).

The findings from Jin et al.‘s (2019), research revealed the critical function of GLUT5 in ccRCC by promoting fructose uptake and utilization, making GLUT5 not only a facilitator of cancer metabolism but also a potential therapeutic target (Jin et al. 2019). This study demonstrated that GLUT5 inhibition, particularly through agents like 2,5-AM, can impede cancer cell proliferation and induce apoptosis, making it a promising approach for ccRCC treatment.

### Intestinal cancers

The GLUT5 transporter plays a significant role in the development and progression of intestinal cancers. High-fructose corn syrup (HFCS), prevalent in many diets, has been shown to enhance tumor growth in the intestines. The study by Goncalves et al. (2019) provides compelling evidence that the consumption of HFCS can increase fructose concentrations in the intestinal lumen and serum, supporting tumor growth in the absence of obesity and metabolic syndrome (Table 1).

GLUT5 is crucial for fructose uptake in intestinal epithelial cells (IECs). The research demonstrated that intestinal tumors express higher levels of GLUT5 compared to normal IECs, enabling efficient fructose transport and metabolism within the tumors. This is particularly important because fructose is converted to fructose-1-phosphate by KHK, which then activates glycolysis and fatty acid synthesis, processes that are essential for tumor growth and survival (Goncalves et al. 2019).

Furthermore, the study found that in genetically modified mice predisposed to develop intestinal tumors (APC mutant mice), the administration of HFCS led to a significant increase in tumor size and grade. This was attributed to the upregulation of GLUT5 and other fructose-metabolizing enzymes within the tumors, highlighting the transporter’s role in facilitating fructose-driven tumorigenesis (Goncalves et al. 2019). The results suggest that targeting GLUT5 or KHK could be a potential therapeutic strategy to inhibit the growth of intestinal cancers driven by fructose metabolism.

### Experimental colitis

Inflammatory bowel disease (IBD), which includes Ulcerative colitis (UC) and Crohn’s disease (CD), represents a spectrum of chronic inflammatory conditions targeting the gastrointestinal tract (Abraham and Cho 2009). The proliferation of IBD has been attributed to the widespread consumption of a western diet, notably rich in fructose. Excessive dietary fructose is linked to its accumulation and colonic microbiota modifications, contributing to the aggravation of experimental colitis (Kawabata et al. 2018; Khan et al. 2020; Montrose et al. 2021). Experiments indicate that fructose feeding in GLUT5-deficient (GLUT5^−/−^) mice results in increased fructose levels and altered colonic microbiota, unlike their GLUT5-deficient or GLUT5^+/+^ counterparts on a fructose or sucrose-free regimen (Basu et al. 2021). Furthermore, the exacerbation of colitis in heterozygous mice, compared to GLUT5^+/+^ mice, points to the detrimental influence of fructose on IBD, mediated mainly by GLUT5 inactivation (Basu et al. 2021). Supporting this, CD patients exhibit lower GLUT5 expression in ileal epithelial cells than healthy controls, alongside an upregulation of proinflammatory cytokine genes such as tumor necrosis factor alpha (*TNF*), interleukin 6 (*IL6*), and interleukin-1 beta (*IL1-β*), indicating an inflammatory cascade triggered by reduced GLUT5 function (Basu et al. 2021). The inverse correlation between GLUT5 expression and proinflammatory cytokines in GLUT5-deficient mice strengthens the hypothesis that inflammatory mediators may suppress GLUT5 expression, warranting further exploration into the relationship between inflammation and GLUT5 in IBD development.

### Colorectal cancer

CRC ranks as the third most prevalent cancer worldwide (Xi and Xu 2021), with an increasing incidence among the younger demographic, necessitating a comprehensive exploration of its underlying drivers and contributing factors.

The role of the GLUT5 transporter in CRC has been the focus of scientific research for over three decades, yet its mechanisms demand further elucidation. Initially, Mahraoui et al. (1992) investigated the GLUT5 transporter in the Caco-2 colon malignant cell line, uncovering its presence at the mRNA and protein levels, marking an early insight into GLUT5’s involvement in CRC (Table 1). Subsequently, Mesonero et al. (1995), delved deeper, analyzing the impact of glucose and fructose on GLUT5 expression within Caco-2 cells. Their findings showed a decrease in GLUT5 expression under hexose-deprived conditions, compared to a significant increase in GLUT5 protein and mRNA levels in cells cultured with fructose, emphasizing the hexose-specific regulation of GLUT5 (Mesonero et al. 1995).

Building on this foundational knowledge, Shen et al. (2022), further outlined GLUT5’s significance, linking its upregulation to increased cell proliferation and resistance to chemotherapy. They demonstrated that CRC cells enriched with fructose upregulated KHK, thereby facilitating the conversion of fructose into fructose-1-phosphate, a critical step in fructose metabolism. This interaction between KHK and GLUT5 catalyzed fructose-driven glycolysis and the tricarboxylic acid cycle, underscoring the metabolic adaptability of malignant CRC cells. Interestingly, silencing KHK attenuated the oncogenic function of GLUT5, suggesting the role of the GLUT5-KHK axis in CRC’s malignant metabolism (Table 1). Shen et al. (2022) proposed that targeting this metabolic pathway through dietary modifications and pharmacological interventions could impede CRC progression.

Further advancing the field, Lin et al. (2021) investigated *SLC2A5* expression in CRC tissues and cell lines and its metastatic implications. Their research unveiled a novel metastatic mechanism mediated by the S100 calcium-binding protein P (S100P), which stimulates the demethylation and activation of *SLC2A5* transcription, thereby elevating GLUT5 protein expression. This breakthrough offers new details into the molecular dynamics facilitating CRC metastasis and highlights the potential of targeting *SLC2A5*/GLUT5 in therapeutic strategies.

Another study investigated the presence of GLUT5, GLUT2, and SGLT1 transporters in the colonic mucosa of healthy individuals and patients with IBD and found them to be ubiquitously expressed in the mucosal epithelial cells of both groups (Merigo et al. 2018). Notably, GLUT5 expression was also observed in lymphatic vessel clusters within these populations, linking its role in the unusual congregation of lymphatic vessels (Merigo et al. 2018). This insight holds potential significance for the histopathological evaluation of lymphangiogenesis in the gastrointestinal tract, particularly regarding cancer and inflammatory bowel diseases, where GLUT5 expression and lymphatic vessel density might serve as early diagnostic markers.

Further research by Włodarczyk et al. (2021), emphasized a fivefold increase of GLUT5 expression in colon cancer tissues compared to healthy colon mucosa. Remarkably, 96.7% of CRC tissues expressed GLUT5, compared to just 53.3% in healthy mucosal samples, with a positive correlation identified between GLUT5 expression and cancer grade. This study also highlighted the efficacy of the GLUT5 inhibitor MSNBA, which significantly reduced colon cancer cell viability and inhibited cancer growth without adversely affecting healthy cells (Fig. 3).

Chałaskiewicz et al. (2023), explored the genetic regulation of GLUT5, discovering that the application of a histone deacetylase inhibitor, trichostatin A, increased *SNAI1* and *SNAI2* expression in high mesenchymal cells, consequently downregulating *SLC2A5* expression. Interestingly, trichostatin A pretreatment made colon cancer cells more susceptible to platinum compounds, addressing a significant challenge in colon cancer treatment (Chałaśkiewicz et al. 2023) (Fig. 3).

Given the considerable demand for glucose in tumorigenesis, which is often unmet due to vascular dysfunction and metabolic competition within tumors, CRC cells notably adapt by utilizing fructose as an alternate energy source. Therefore, while the specific mechanisms of this metabolic compensation ask for further investigation, targeting fructose metabolism via GLUT5 regulation emerges as a promising approach for developing novel CRC therapeutics.

## Future directions: unraveling mechanisms and therapeutic approaches

Investigating GLUT5’s role in cancer biology and its tumorigenic implications, including effects on proliferation, apoptosis, migration, and invasion, offers an opportunity to deepen our understanding of cancer mechanisms and develop new therapeutic strategies. It is essential to enhance the research on how GLUT5 overexpression alters cancer cell metabolism, causing changes in energy production, biosynthesis pathways, and redox balance due to altered fructose metabolism. Assessing the influence of GLUT5 overexpression on the tumor microenvironment and its role in tumor immune evasion and exploring it as a diagnostic, prognostic, or early detection biomarker could revolutionize patient management, therapy selection, and outcome prediction. Comprehensive analysis of the genetic and epigenetic landscape in tumors overexpressing GLUT5 can identify key regulatory mechanisms controlling GLUT5 expression. This could lead to developing therapies that specifically target GLUT5-overexpressing tumors, including small-molecule inhibitors and monoclonal antibodies. Part of the process of designing effective treatment strategies would be addressing GLUT5 overexpression-related resistance against targeted therapies, as well as the potential of combining GLUT5-targeted interventions with existing treatments to enhance overall efficacy.

Finally, translating preclinical findings into a clinical setting would require trials to assess the safety and effectiveness of GLUT5-targeted therapies. Advancing these research directions requires collaboration among scientists, clinicians, and industry partners to translate scientific discoveries into practical clinical benefits for patients with GLUT5-overexpressing tumors.

## Conclusion

GLUT5 overexpression and its related tumorigenic implications represent a complex and intriguing area of research in cancer biology. It is now evident that GLUT5 plays an important role in the development and progression of various types of tumors. Studies have shown that GLUT5 overexpression can enhance the uptake of fructose by cancer cells, providing them with an alternative energy source and promoting their growth and survival. Additionally, increased GLUT5 expression has been associated with alterations in cellular metabolism, contributing to the Warburg effect, a typical feature of cancer cells characterized by increased glycolysis. These metabolic changes can give a selective advantage to cancer cells, stimulating their proliferation and metastasis. Furthermore, the relationship between GLUT5 overexpression and tumorigenesis extends beyond metabolic adaptations. Recent research suggests that GLUT5 may also be involved in immune evasion mechanisms employed by cancer cells, thus further promoting tumor growth. Future research should focus on elucidating the precise molecular pathways and regulatory mechanisms driving GLUT5 overexpression in different cancer types, understanding GLUT5’s role in fructose-related metabolic disorders, exploring pharmacological interventions, and translating these findings into practical applications for improved healthcare.

## Data Availability

Not applicable.

## Abbreviations

1-FDAM: 1-deoxy-1-fluoro-2,5-anhydro-D-mannitol
2,5-AM: C-3-modified 2,5-anhydro-*D*-mannitol
6-[^18^F]FDF: [^18^F]-labeled 6-deoxy-6-fluoro-D-fructose
12-LOX: 12-Lipoxygenase
12-HETE: 12-Hydroxy-5Z,8Z,10E,14Z-eicosatetraenoic acid
^18^F-FDG: [¹⁸F]-Fluorodeoxyglucose
^18^F-FDG PET-CT: [^18^F]-Fluorodeoxyglucose positron emission tomography-computed tomography
ALDOB: Aldolase B
AML: Acute myeloid leukemia
ATP: Adenosine triphosphate
BC: Breast cancer
CaP: Prostate cancer
CCA: Cholangiocarcinoma
ccRCC: Clear cell renal cell carcinoma
CD: Crohn’s disease
CDH1: Cadherin 1
CLB: Chlorambucil
CRC: Colorectal cancer
DHAP: Dihydroxyacetone phosphate
EMT: Epithelial-to-mesenchymal transition
GA: Glyceraldehyde
GLUT: Glucose transporter
HFCS: High fructose corn syrup
HGPIN: High-grade prostatic intraepithelial neoplasia
HIF1α: Hypoxia-inducible factor 1 alpha
HK2: Hexokinase 2
IBD: Inflammatory bowel disease
IECs: Intestinal epithelial cells
IL6: Interleukin 6 (gene)
IL1β: Interleukin-1 beta (gene)
KHK: Ketohexokinase
LC: Lung carcinoma
LDHA: Lactate dehydrogenase A
LUAD: Lung adenocarcinoma
MCT4: Monocarboxylate transporter 4
MRI: Magnetic resonance imaging
MSNBA: N-[4-(methylsulfonyl)-2-nitrophenyl]-1,3-benzodioxol-5-amine
NBD: 7-Nitro-1,2,3-benzoxadiazole
NSCLC: Non-small-cell lung carcinoma
OC: Ovarian carcinoma
OSCC: Oral squamous cell carcinoma
PET: Positron emission tomography
PCL-PEG-Fru: D-fructose-modified poly(ε-caprolactone)-polyethylene glycol
PPF MM: PCL-PEG-Fru/TPGS mixed micelles
RGD: Arginylglycylaspartic acid
S100P: S100 calcium-binding protein P
SCLC: Small-cell lung carcinoma
SLC2A: Solute carrier family 2 A
SNAI2: Snail family transcriptional repressor 2
SPECT: Single-photon emission computed tomography
TK: Triose kinase
TNF: Tumor necrosis factor alpha (gene)
TPGS: D-α-tocopheryl polyethylene glycol 1000 succinate
UC: Ulcerative colitis
YWHAH: Tyrosine 3-monooxygenase/tryptophan 5-monooxygenase activation protein eta
